# Risk of symptomatic gallstones and cholecystectomy after a very-low-calorie diet or low-calorie diet in a commercial weight loss program: 1-year matched cohort study

**DOI:** 10.1038/ijo.2013.83

**Published:** 2013-06-18

**Authors:** K Johansson, J Sundström, C Marcus, E Hemmingsson, M Neovius

**Affiliations:** 1 Clinical Epidemiology Unit, Department of Medicine (Solna) Karolinska Institutet, Stockholm, Sweden; 2 Uppsala University, Department of Medical Sciences, Uppsala, Sweden; 3 Karolinska Institutet, Department of Clinical Science, Intervention and Technology (CLINTEC), Stockholm, Sweden; 4 Karolinska Institutet, Obesity Center, Department of Medicine, Stockholm, Sweden

**Keywords:** VLCD, LCD, commercial weight loss, gallstones, cholecystectomy, adverse events

## Abstract

**Background:**

Concern exists regarding gallstones as an adverse event of very-low-calorie diets (VLCDs; <800 kcal per day).

**Objective:**

To assess the risk of symptomatic gallstones requiring hospital care and/or cholecystectomy in a commercial weight loss program using VLCD or low-calorie diet (LCD).

**Design:**

A 1-year matched cohort study of consecutively enrolled adults in a commercial weight loss program conducted at 28 Swedish centers between 2006 and 2009. A 3-month weight loss phase of VLCD (500 kcal per day) or LCD (1200–1500 kcal per day) was followed by a 9-month weight maintenance phase. Matching (1:1) was performed by age, sex, body mass index, waist circumference and gallstone history (*n*=3320:3320). Gallstone and cholecystectomy data were retrieved from the Swedish National Patient Register.

**Results:**

One-year weight loss was greater in the VLCD than in the LCD group (−11.1 versus −8.1 kg; adjusted difference, −2.8 kg, 95% CI −3.1 to −2.4; *P<*0.001). During 6361 person–years, 48 and 14 gallstones requiring hospital care occurred in the VLCD and LCD groups, respectively, (152 versus 44/10 000 person–years; hazard ratio, 3.4, 95% CI 1.8–6.3; *P<*0.001; number-needed-to-harm, 92, 95% CI 63–168; *P<*0.001). Of the 62 gallstone events, 38 (61%) resulted in cholecystectomy (29 versus 9; hazard ratio, 3.2, 95% CI 1.5–6.8; *P*=0.003; number-needed-to-harm, 151, 95% CI 94–377; *P<*0.001). Adjusting for 3-month weight loss attenuated the hazard ratios, but the risk remained higher with VLCD than LCD for gallstones (2.5, 95% CI 1.3–5.1; *P=*0.009) and became borderline for cholecystectomy (2.2, 95% CI 0.9–5.2; *P=*0.08).

**Conclusion:**

The risk of symptomatic gallstones requiring hospitalization or cholecystectomy, albeit low, was 3-fold greater with VLCD than LCD during the 1-year commercial weight loss program.

## Introduction

Bariatric surgery is currently the most effective treatment for obesity,^1^ but all obese patients cannot undergo surgery because of guidelines, contraindications, capacity constraints and patient preferences. With the limited capacity of hospital-based obesity treatment, commercial weight loss programs including intensive treatment such as very-low-calorie diets (VLCDs; defined as <800 kcal per day) ^2^ are used by millions each year.^2, 3, 4^

The effects of commercial weight loss programs have shown to equal or even outweigh primary-care programs,^5^ with initial rapid weight loss using VLCD compared with low-calorie diet program (LCD; 1200–1500 kcal per day) inducing greater 1-year weight losses and lower dropout rates.^6^ However, the safety of commercial programs has rarely been studied, and using VLCD in commercial weight loss programs is a particular concern given the risk of adverse events as well as weight regain.^7, 8, 9^

A commonly described adverse event after rapid weight loss (including VLCD) is gallstones. Previous studies of VLCD-induced rapid weight loss have generally been small, of short duration, lacked control groups and used formulations with lower amounts of fat compared with the formulations used today.^10, 11, 12, 13, 14^ These previous studies have all identified gallstone formation with ultrasonography after VLCD use. However, the majority of the gallstones found in these studies were asymptomatic, and few led to symptoms. The risk of symptomatic gallstones requiring hospital care and/or cholecystectomy, potentially serious adverse events after VLCD use, has not been studied. Further, to the best of our knowledge, there are no large-scale studies on the safety of commercial weight loss programs.

The aim of this matched cohort study was to investigate in a real-life setting the risk of symptomatic gallstones leading to hospitalization or cholecystectomy after using VLCD (500 kcal per day for 6–10 weeks) compared with participants on LCD (1200–1500 kcal per day for up to 3 months) during a 1-year commercial weight loss program.

## Methods

**Figure 1:**
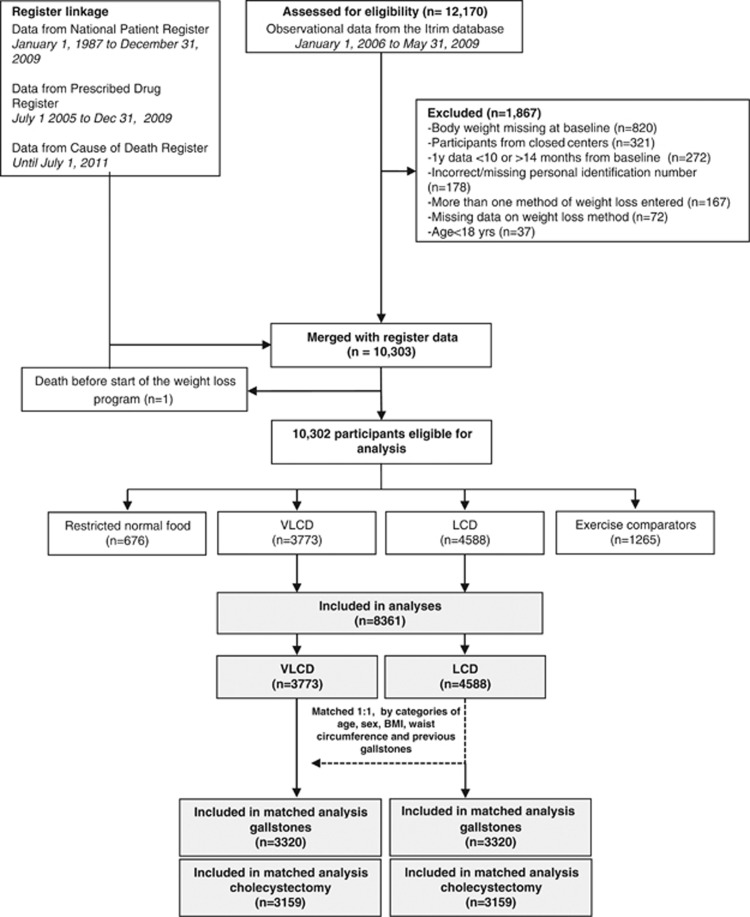
Flow chart of included participants and matching.

This matched cohort study was conducted in the commercial weight loss setting in Sweden. Participant data were retrieved from the database used by the commercial company to track customer progress and compliance. These data were linked to the National Patient Register,^15^ containing inpatient and nonprimary outpatient care, for outcome ascertainment, and to the Causes of Death Register for follow-up of vital status (Figure 1). The regional Ethics Committee in Stockholm, Sweden, approved the study.

### Participants

Included participants were consecutively enrolled adult customers (age ⩾18years; *n*=8361) from the commercial weight loss company Itrim in Sweden (www.itrim.se) from 1 January 2006 to 31 May 2009. Data were collected from 28 centers across Sweden, as described elsewhere.^6^

### Interventions

The weight loss program was of 1-year duration and comprised an initial 3-month weight loss phase, followed by a 9-month weight maintenance phase. At enrollment, participants selected one of three programs (VLCD, LCD or normal food, as described elsewhere^6^). In this study, we report data from the VLCD and LCD groups, as these are the two programs including liquid formula diets. Although all participants were paying customers and were free to choose weight loss method, the company used criteria for VLCD use, similar to the recommendations in the European (Scientific Co-operation ) SCOOP-report on VLCD use (Supplementary Table 1).^16^ VLCD participants needed to sign a form, where they had been informed that VLCD use carries an increased risk of the adverse events listed in the SCOOP-report on VLCD use.^16^

#### Weight loss phase (0–3 months)

*VLCD*: Liquid-based formula diet of 500 kcal per day for 6–10 weeks (Itrim, Stockholm, Sweden; 125 kcal per sachet, 4 sachets per day, each sachet contained 13 *g* protein, 15 *g* carbohydrates, 2 *g* fat and 3 *g* fiber; approved as sole source VLCD by the Swedish National Food Agency), followed by a 2-week gradual introduction of normal food. Early introduction of normal food (6 as opposed to the full 10 weeks) occurred when the participant was either satisfied with the achieved weight loss or had reached a body mass index (BMI) <25.0 kg m^−2^.

*LCD*: consisting of two calorie-restricted normal food meals and two formula diet meal replacement sachets (á 125 kcal), providing a total caloric intake of ∼1200–1500 kcal per day depending on body size and exercise levels. The normal food consisted of restricted portion sizes with a low overall energy density, high in protein, and with a low-glycemic index.^6^

#### Weight maintenance phase (3–12 months)

After the weight loss phase, the two groups entered the same 9-month weight maintenance program that included an exercise program (circuit training, with a mix of aerobic and strength training work-out stations, at the center 2–3 times per week for 30–45 min, physically active transport to and from work and using a Yamax SW-200 pedometer to encourage walking), dietary advice, self-monitoring and behavioral changes. Dietary advice included the use of restricted portion sizes, and eating a diet rich in protein and with a low-glycemic index, with a low overall energy density.^6^

### Data collection

#### Participant data

Anthropometric data were collected by company-trained health coaches at baseline, 3, 6 and 12 months, and recorded into the database of the commercial company. Body weight was measured in a nonfasting state with the Tanita TBF-300 bioelectrical impedance monitor (Tanita Corporation, Tokyo, Japan). Waist circumference was measured midway between the iliac crest and the lower rib cage (exhaled) with a measuring tape and was classified as normal (<80 cm for women/<94 cm for men), increased risk (80–87/94–101 cm) and high risk (⩾88/102 cm).^17^ Height (without shoes) was measured by a wall-mounted stadiometer. World Health Organization BMI criteria (kg m^−2^) were used to classify participants as underweight (<18.5), normal weight (18.5–24.9), overweight (25.0–29.9) and obese class I/II/III (30.0–34.9/35.0–39.9/⩾40).^18^

#### Register data

Data on gallstones requiring hospital care, cholecystectomy, mortality and comorbidity were retrieved from National registers via register linkage. In Sweden, all the residents have a 10-digit personal identification number recorded in the medical files and nationwide health and census registers, enabling deterministic linkage.

#### History of gallstones, comorbidity and drug use

Data on the history of gallstones requiring hospital care during the 5 years before program were collected from the National Patient Register, which contains nationwide data on inpatient and nonprimary outpatient care in Sweden. Cholecystectomy history was retrieved from the same source from 1987 and onward. Data on hospital visits for malignancies (International Classification of Diseases [ICD] version 10 codes C00–C97) and circulatory disease (I00–I99) were also collected.

Drug dispensation data were retrieved from the nationwide Prescribed Drug Register during the 6 months preceding program start for antidiabetic drugs (Anatomical Therapeutic Classification [ATC] classification system codes A10A and A10B), antihypertensive drugs (C02, C03, C07, C08, C09), lipid-lowering drugs (C10AA, C10AB, C10AC, C10AD, C10B, C10AX), antidepressants (N06A), the antiobesity drugs orlistat (A08AB01), sibutramine (A08AA10) and rimonabant (A08AA11) and ursodeoxycholic acid (A05A).

#### Outcome and follow-up data

The primary outcome was gallstones requiring hospital care (cholelithiasis, ICD10 code K80). Cholecystectomy (procedure codes JKA20/JKA21) and all-cause mortality were investigated as secondary outcomes. Participants were followed from program start until first event, death, program end or 31 December, 2009, whichever came first. Cholecystectomized patients were excluded in the analysis of cholecystectomy but included in the analysis of gallstones requiring hospital care, as gallstones still can form in the gall ducts.

Gallstone and cholecystectomy data were retrieved from the National Patient Register.^15^ Mortality data were retrieved from the Causes of Death Register, which contains information on >99% of all deaths in Sweden.^19^

### Statistical analysis

LCD participants were matched with replacement to VLCD participants 1:1 by age (+/−1-year), sex, BMI category, waist circumference category and previous gallstones. Analyses included all matched patients and were analyzed by intention to treat.

Kaplan–Meier curves were constructed to illustrate the absolute risk. To compare the risk of gallstones and cholecystectomy between the programs, we used conditional Cox regression to estimate hazard ratios over the 1-year follow-up (conditioned on the matching factors and adjusted for factors with significant differences at baseline).

A sensitivity analysis was conducted restricting the study population to participants without gallstones requiring hospital care during the 5 years preceding program start. Mortality was not analyzed due to few cases (one death due to unknown cause occurred in the VLCD group after the weight loss phase).

An exploratory analysis, combining the two intervention groups, was also conducted using multivariable Cox regression to investigate factors potentially associated with gallstones requiring hospital care, or cholecystectomy, including age, sex, baseline BMI, history of gallstones and weight loss during the rapid weight loss phase (0–3 months).

Data were analyzed using SAS (version 9.3). All reported *P*-values are two-sided and *P*-values <0.05 were regarded as statistically significant.

## Results

After matching LCD participants (*n*=4588) with replacement to VLCD participants (*n*=3773) 1:1 by age, sex, BMI, waist circumference, and previous gallstones, 3320 participants remained in each group (Figure 1).

### Baseline characteristics

At baseline, the mean age was 46 years, mean BMI 33.4 kg m^−2^ and 83% were women. Fifty-one percent of the participants were class I obese, 23% were class II obese, 8% were class III obese and 19% were overweight.

The two treatment groups were balanced regarding circulatory disease, malignancy history and the use of lipid-lowering drugs and antidepressants. There were fewer users in the VLCD than in the LCD group of antidiabetes drugs (1.8 versus 3.3% mean difference, −1.4%, 95% CI −0.7 to −2.2% *P<*0.001) and antihypertensives (16.7% versus 19.4% mean difference, −2.7%, 95% CI −0.8 to −4.5% *P*=0.005; Table 1). At baseline, 0.9% (*n*=58/6640) had a history of gallstones requiring hospital care, of which 74% (*n*=43/58) had been cholecystectomized (Table 1).

### Weight change

**Figure 2:**
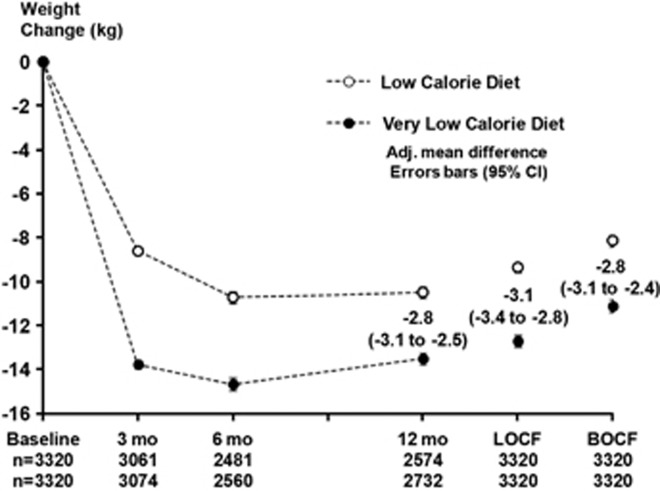
Weight change during the 1-year program LOCF, last observation carried forward (to 12 months); BOCF, baseline observation carried forward (to 12 months).

Eighty-two percent of the VLCD group and 78% in the LCD group completed the 1-year program (odds ratio, 1.3, 95% CI 1.2–1.5; *P<*0.001). After the initial weight loss phase (0–3 months; baseline observation carried forward), weight loss was 12.7 versus 7.9 kg (adjusted mean difference, 4.6, 95% CI 4.4–4.9; *P<*0.001). After the entire 1-year program, weight loss was 11.1 versus 8.1 kg (adjusted mean difference, 2.8, 95% CI 2.4–3.1; *P<*0.001; Figure 2). Data for the unmatched population have been described elsewhere.^6^

### Risk of gallstones requiring hospital care

**Figure 3:**
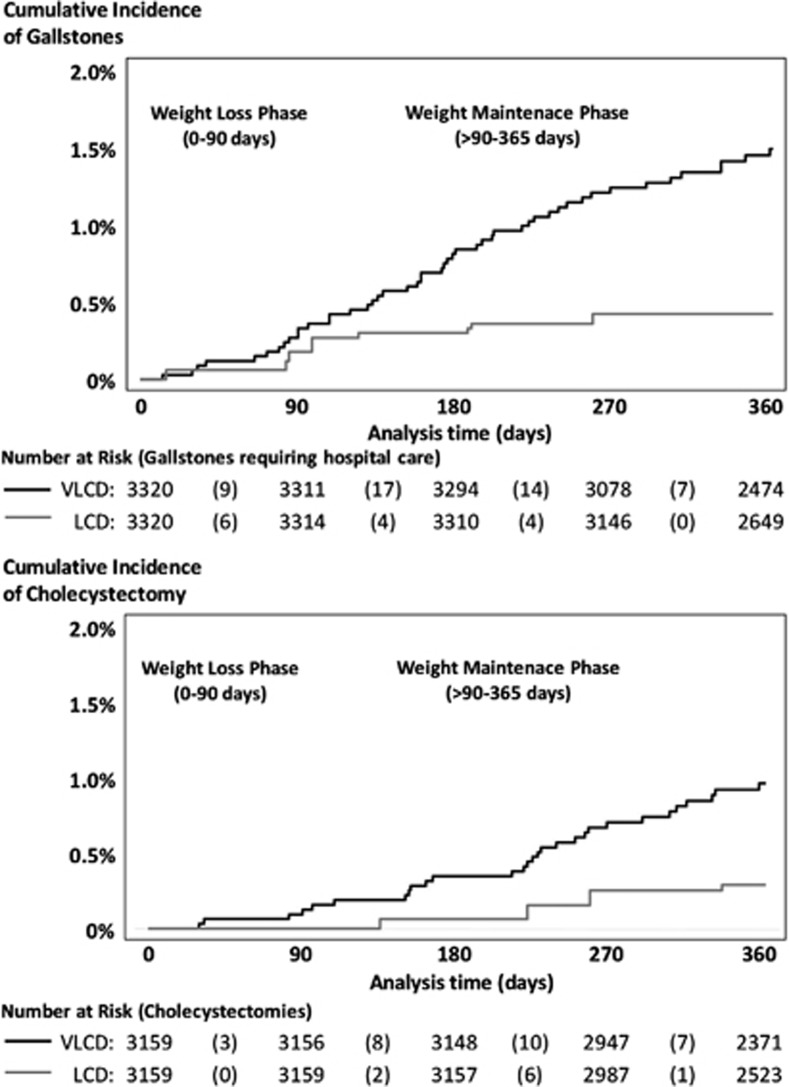
Risk of gallstones requiring hospital care (upper panel) and cholecystectomy (lower panel) by weight loss program. VLCD, very-low-calorie diet; LCD, low calorie diet.

During 6361 person–years of follow-up, 48 gallstones occurred in the VLCD group and 14 in the LCD group (152 versus 44 per 10 000 person–years; conditional hazard ratio, 3.4, 95% CI 1.8−6.3; *P<*0.001; Figure 3; Table 2). The risk difference was 108 per 10 000 person–years (95% CI 59–157; *P<*0.001), resulting in a number-needed-to-harm of 92 (95% CI 63–168; *P<*0.001). Adjusting the main analysis for weight loss during the first 3 months attenuated the hazard ratio, but the hazard ratio remained higher with VLCD than LCD (2.5, 95% CI 1.3–5.1; *P<*0.001).

### Risk of cholecystectomy

Including only participants who did not undergo a cholecystectomy preceding program start (*n*=3159 in the VLCD group and 3159 in the LCD group), 38 cases of cholecystectomy were performed during 6067 person–years of follow-up, of which 29 were in the VLCD group and 9 in the LCD group (96 versus 30 per 10 000 person–years; conditional hazard ratio, 3.2, 95% CI 1.5–6.8; *P*=0.003; number-needed-to-harm, 151, 95% CI 94–377; *P<*0.001; Figure 3; Table 2). Adjustment for weight loss during the first 3 months attenuated the hazard ratio (2.2, 95% CI 0.9–5.2; *P*=0.08).

### Sensitivity analysis

Excluding participants with gallstones requiring hospital care during the 5 years preceding program start (*n*=58) resulted in similar results for gallstones (46 versus 13 in the VLCD and LCD group, respectively; 147 versus 41 per 10 000 person–years; hazard ratio, 3.4, 95% CI 1.8–6.3; number-needed-to-harm, 94, 95% CI 65–173; *P<*0.001) and cholecystectomy (28 in the VLCD group compared with 9 in the LCD group; 93 versus 30 per 10 000 person–years; hazard ratio, 3.2, 95% CI 1.5–6.8; number-needed-to-harm, 158, 95% CI 97–420; *P*=0.002).

### Exploratory analysis: predictors of gallstones and cholecystectomy during follow-up

In multivariable analysis, the risk of developing gallstones requiring hospital care was higher in women than in men, in younger than in older participants, in those with a higher baseline BMI, among those who lost the most weight and in those with a history of gallstones (irrespective of cholecystectomy status; Supplementary Table 2). For cholecystectomy, the same factors as for gallstones requiring care were associated with an increased risk for cholecystectomy (Supplementary Table 3).

## Discussion

In our analysis of symptomatic gallstones requiring hospital care or cholecystectomy in participants using either VLCD or LCD for the first 3 months of a 1-year commercial weight loss program, we found the absolute risk of gallstones as well as cholecystectomy to be low but approximately three times higher in the VLCD than in the LCD group. After adjusting for weight loss during the first 3 months, the risk was attenuated but remained higher with VLCD than LCD, suggesting a direct effect of VLCD on gallstone disease. To the best of our knowledge, this is the largest controlled study of VLCD and of the risk of severe gallstone problems and the first large-scale safety analysis of a commercial program.

### Previous research

Previous studies have investigated the association between VLCD and ultrasonography-assessed gallstone formation, rather than the risk of gallstones as a serious adverse event requiring hospital care and/or cholecystectomy. The majority of these studies were conducted in the late 1980s and early 1990s using VLCDs containing low levels of fat (≈1g per day).^10, 11, 12, 13, 14^ In a review of these studies,^20^ Everhart reported that 10–25% of VLCD participants developed gallstones, one-third of which were symptomatic. Limitations of these studies were the lack of control groups, small sample sizes and short follow-up (8–36 weeks).^10, 11, 12, 13, 14^ Later studies included VLCDs containing higher fat content (12–30 g per day),^21, 22, 23, 24^ two of which were randomized controlled trials.^21, 22^ None developed systematic gallstones in the high-fat group in either of the studies, suggesting that an adequate fat intake reduces gallstone formation. The fat content of the VLCD in our study was 7–9 g per day, consistent with the 7 g per day recommendation given by the European SCOOP-report on VLCD use.^16^ The higher risk with VLCD in our study suggests that fat content may need to be increased even further to reduce the risk to LCD levels.

### Mechanisms

Increased risk for gallstone formation during VLCDs could be explained by inadequate fat content of the diet and/or the rapid weight loss associated with VLCDs. Rapid weight loss, either by VLCD or bariatric surgery, is a known risk factor for gallstone formation.^20^ The two most commonly suggested mechanisms for gallstone formation are supersaturation of bile with cholesterol, leading to cholesterol crystallization and stone formation, and the insufficient gallbladder emptying due to impaired motility.^25^ Rapid weight loss induced by VLCDs is believed to affect both the mechanisms: Supersaturation is believed to be caused by decreased bile salt levels and increased cholesterol levels, and impaired motility due to reduced gallbladder stimulation because of the low-fat content.^26, 27^ However, as described previously, a fat intake of 7–10 g per day has been reported as a threshold for maintaining an efficient gallbladder emptying.^16, 26^ The majority of the gallstones requiring hospital care occurred during the maintenance phase (37/48 in the VLCD group and 8/14 in the LCD group). This was also the case in a previous study of VLCD as a treatment for sleep apnea (3/3 gallstones reported during the maintenance phase).^28, 29^

### Clinical implications

Increased risk of gallstone formation during and after VLCD-induced rapid weight loss is incorporated in clinical recommendations as an adverse event, advising physicians to inform patients about this risk, but the risk magnitude has been unclear.^7, 16, 20^ Our findings indicate a small, absolute but substantially elevated relative risk of gallstones requiring hospital care and/or cholecystectomy when using VLCD instead of LCD.

Whether the benefits of the additional weight loss in the VLCD group are worth the extra risk for gallstones and cholecystectomy may depend on patients' disease and risk factor status, as well as their preferences. Supplementation with omega-3 fatty acids and/or the use of ursodeoxycholic acid during the rapid weight loss phase could possibly reduce gallstone formation.^26, 30^

### Strengths and limitations

The strengths of the current study include the large sample of weight loss participants in a real-life setting, with a direct VLCD and LCD comparison. With the risk of symptomatic gallstones being low, statistical power may become an issue in smaller studies. Our large study made it possible to study the risk of symptomatic gallstones leading to hospitalization and/or cholecystectomy as a serious adverse event to VLCD. Further, the outcomes were prospectively reported and data were collected routinely on a nationwide level in the universally accessible Swedish health-care system, with virtually complete follow-up.

The main limitation was the nonrandomized design. Baseline differences in age, sex, BMI, waist circumference and gallstone history were handled by matching. Multivariable adjustment was used for handling remaining baseline imbalances. However, residual confounding may exist. The results appeared to be robust in our sensitivity analyses.

Second, participants had selected and paid for the treatment themselves, possibly limiting generalizability to the wider overweight and obese population. Finally, the National Patient Register contains data on inpatient and nonprimary outpatient visits for gallstones, not visits in primary care, or undetected asymptomatic gallstones. Our results may therefore not be generalizable to mild or asymptomatic gallstones. However, our primary outcomes were gallstones requiring hospital care and cholecystectomies. Gallstones treated in the primary care, at home, or not treated at all, are likely both less serious for the patient and less costly for society.

## Conclusion

The absolute risk of gallstones as well as cholecystectomy was found to be low but approximately three times higher in the VLCD than in the LCD group during the 1-year commercial weight loss program. After adjusting for weight loss during the first 3 months, the risk was attenuated but remained higher with VLCD than LCD, suggesting a direct effect of VLCD on gallstone disease.

## Figures and Tables

**Table 1:** Baseline characteristics of matched participants enrolled in a commercial weight loss program using very-low-calorie diet or low-calorie diet

	*Very-low-calorie diet (*n*=3320)*	*Low-calorie diet (*n*=3320)*	*P-value*^a^
Women	2764 (83%)	2764 (83%)	1.00
Age (years)	46 (11),18–75	46 (11),18–76	0.85
			
*Body weight (kg)*			
Women	93 (13), 64–168	92 (15), 59–190	0.002
Men	111 (14), 77–180	109 (15), 77–188	0.003
			
*BMI (kg m*^*−2*^)			
18.5–24.9	13 (0%)	13 (0%)	1.00
25–29.9	616 (19%)	616 (19%)	
30–34.9	1692 (51%)	1692 (51%)	
35–39.9	748 (23%)	748 (23%)	
⩾40	251 (8%)	251 (8%)	
			
*Waist circumference (cm)*			

*Women*			
80–87	37 (1%)	37 (1%)	1.00
⩾88	2615 (95%)	2615 (95%)	
			
*Men*			
94–101	2 (0%)	2 (0%)	1.00
⩾102	541 (97%)	541 (97%)	
			
*Event history*			
Gallstones (last 5 years)	29 (0.9%)	29 (0.9%)	1.00
Cholecystectomy (since 1987)	137 (4.1%)	180 (5.4%)	0.01
			
*Comorbidity history*			
Circulatory Disorders	267 (8.0%)	272 (8.2%)	0.82
Malignancy	54 (1.6%)	70 (2.1%)	0.15
			
*Drug use (last 6 months)*^b^			
*Any antidiabetes drug*	61 (1.8%)	108 (3.3%)	<0.001
Insulin	12 (0.4%)	43 (1.3%)	<0.001
Oral antidiabetics	56 (1.7%)	93 (2.8%)	0.002
Lipid-lowering agents	195 (5.9%)	217 (6.5%)	0.26
Antihypertensives	556 (16.7%)	645 (19.4%)	0.005
*Any antiobesity drug*	87 (2.6%)	82 (2.5%)	0.70
Orlistat	31 (0.9%)	38 (1.1%)	0.40
Sibutramine	56 (1.7%)	49 (1.5%)	0.49
Rimonabant	0 (0.0%)	0 (0.0%)	1.00
Antidepressants	391 (11.8%)	432 (13.0%)	0.13
Ursodeoxycholic acid	0 (0.0%)	2 (0.1%)	0.16

Data for continuous variables are mean (s.d.), min–max, and n (%) for categorical variables.

a *P*-values are from independent samples *t*-tests for continuous variables and from *χ*^2^ tests for categorical variables. Waist circumference was missing for *n*=348 (5%). N=124 (4%) for VLCD and N=124 (4%) for LCD.

b Drug use during the last 6 months was assessed via register linkage to the Prescribed Drug Register, while comorbidity, gallstones and cholecystectomies were retrieved from the National Patient Register during the last 5 years (comorbidity and gallstones), or from 1987 and onwards (cholecystectomy history).

**Table 2:** Risk of gallstones requiring hospital care and cholecystectomy

	*Participants at risk (*n)	*Observation years (sum)*	*Events (*n)	*Events/10 000 person–years (95% CI)*	*Hazard ratio* *(95% CI)*	*Hazard ratio*^a^ *(95% CI) additionally adjusted for 3-month weight loss*
*Gallstones requiring hospital care*						
VLCD	3320	3163	48	152 (110–194)	3.4 (1.8–6.3) *P<0.001*	2.5 (1.3–5.1) *P=0.009*
LCD	3320	3198	14	44 (21–66)	1.0 (ref.)	1.0 (ref.)
						
*Cholecystectomy*						
VLCD	3159	3021	29	96 (62–130)	3.2 (1.5–6.8) *P=0.003*	2.2 (0.9–5.2) *P=0.08*
LCD	3159	3046	9	30 (11–48)	1.0 (ref.)	1.0 (ref.)

Abbreviations: VLCD, very-low calorie diet; LCD, low-calorie diet.

## Notes

All authors have completed ICMJE Form for Disclosure of Potential Conflicts of Interest. Dr Hemmingsson report that he has received consultancy fees from Itrim and was employed part-time by Itrim as program director during 2006-2008. Drs Neovius, Sundström and Marcus report that they are members of Itrim's Scientific Advisory Board. Dr Johansson has no financial relationships with Itrim, but received a grant from Cambridge Weight Plan, Northants, (manufacturer of another VLCD formula) for two studies examining the effect of weight loss on obstructive sleep apnea.^28, 29^
