# Supplementary material for: Risk of symptomatic gallstones and cholecystectomy after a very-low-calorie diet or low-calorie diet in a commercial weight loss program: 1-year matched cohort study
Source: Int J Obes (Lond). 2013 Jun 18;38(2):279–84. doi: 10.1038/ijo.2013.83 (PMC3921672; doi:10.1038/ijo.2013.83)
Supplement: Supplementary Information [file ijo201383x1.doc]

**Supplementary information**

**Risk of symptomatic gallstones and cholecystectomy after very low calorie diet or low calorie diet in a commercial weight loss program: 1-year matched cohort study**

**Kari Johansson, PhD, Johan Sundström, MD/PhD, Claude Marcus, MD/PhD, Erik Hemmingsson, PhD, & Martin Neovius, PhD**

**Table of Contents**

**eTable 1** Company criteria and contraindications for VLCD use Page 2

**eTable 2** Predictors of gallstones requiring hospital care Page 3

**eTable 3** Predictors of cholecystectomy Page 4

**eTable 1** Company criteria and contraindications for VLCD use

| **Company Criteria[[1]](#footnote-2)** |
| --- |
| - **BMI≥30 kg/m^2^** - **BMI≥27 kg/m^2^ with elevated waist circumference** (≥102 cm for men and ≥88 cm for women) |
|  |
| **Contraindications[[2]](#footnote-3) -** unless signed approval from physician |
| - insulin-treated diabetes |
| - gallstones not progressed to cholecystectomy during the last 2 years |
| - gout |
| - cancer during the last 2 years |
| - cardiovascular disease during the last 3 months |
| - pregnancy |
| - breastfeeding |
| - catabolic disease |
| - kidney disease |
| - anorexia nervosa or bulimia |
| **Medication During the Program** |
| Participants taking specific drugs were also informed that they needed a physician health examination within three weeks of starting VLCD |

**eTable 2** Predictors of gallstones requiring hospital care

|  | **At Risk**  **(n)** | **Events**  **(n)** | **Multivariable  Hazard Ratio (95%CI)** | **P-Value** |
| --- | --- | --- | --- | --- |
| **Sex** | | | | |
| Women | 5,528 | 60 | 5.4 (1.3-22.3) | *.02* |
| Men | 1,112 | 2 | 1.0 (ref) |
| **Age (Years)** | | | | |
| 1^st^ tertile  *18-40.9y* | 2,213 | 30 | 2.7 (1.3-5.6) | *.01* |
| 2^nd^ tertile  *41-41.5y* | 2,214 | 21 | 2.1 (1.0-4.5) | *.06* |
| 3^rd^ tertile  *51.4-76.1y* | 2,213 | 11 | 1.0 (ref) |  |
| **Baseline BMI** | | | | |
| 1^st^ tertile  *22.5-31.2* | 2,219 | 15 | 1.0 (ref) |  |
| 2^nd^ tertile  *31.3-34.5* | 2,206 | 17 | 1.1 (0.6-2.3) | *.73* |
| 3^rd^ tertile  *34.6-68.1* | 2,215 | 30 | 1.9 (1.0-3.6) | *.06* |
| **Weight Loss 0-3 Months** | | | | |
| 1^st^ tertile  *+9.5 to -8.4kg* | 505 | 4 | 1.0 (ref) |  |
| 2^nd^ tertile  *-8.5 to -13.5kg* | 2,052 | 13 | 1.4 (0.7-3.0) | *.40* |
| 3^rd^ tertile  *-13.6 to -46.1kg* | 2,042 | 16 | 2.2 (1.1-4.4) | *.02* |
| *Missing* | 2,041 | 29 | 1.3 (0.4-4.2) | *.63* |
| **Gallstone History (Last 5 Years)** | | | | |
| Yes | 58 | 3 | 6.6 (1.9-22.7) | *.003* |
| No | 6,582 | 59 | 1.0 (ref) |

**eTable 3** Predictors of cholecystectomy

|  | **At Risk**  **(n)** | **Events**  **(n)** | **Multivariable  Hazard Ratio (95%CI)** | **P-Value** |
| --- | --- | --- | --- | --- |
| **Sex[[3]](#footnote-4)** | | | | |
| Women | 5,218 | 38 | - | - |
| Men | 1,100 | 0 | - |
| **Age (Years)** | | | | |
| 1^st^ tertile  *18-40.7y* | 2,085 | 21 | 3.5 (1.3-9.5) | *.01* |
| 2^nd^ tertile  *40.8-51.4y* | 2,095 | 12 | 2.6 (0.9-7.4) | *.08* |
| 3^rd^ tertile  *51.5-75.4y* | 2,100 | 5 | 1.0 (ref) |  |
| **Baseline BMI** | | | | |
| 1^st^ tertile  *22.5-31.1* | 2,092 | 8 | 1.0 (ref) |  |
| 2^nd^ tertile  *31.2-34.3* | 2,107 | 6 | 0.7 (0.2-2.0) | *.46* |
| 3^rd^ tertile  *34.4-68.1* | 2,081 | 24 | 2.4 (1.1-5.6) | *.03* |
| **Weight Loss During the First 3 Months** | | | | |
| 1^st^ tertile  *+9.5 to -8.4kg* | 1,961 | 8 | 1.0 (ref) |  |
| 2^nd^ tertile  *-8.5 to -13.4kg* | 1,919 | 7 | 0.9 (0.3-2.5) | *.81* |
| 3^rd^ tertile  *-13.5 to -46.1kg* | 1,964 | 21 | 2.4 (1.0-5.8) | *.04* |
| *Missing* | 474 | 2 | 0.8 (0.2-3.8) | *.76* |

1. Company criteria version for the period 2006-2009 [↑](#footnote-ref-2)
2. A health questionnaire completed by the participants before program start was used to detect these contraindications [↑](#footnote-ref-3)
3. Sex not included in the model because of 0 events in men [↑](#footnote-ref-4)
